# Cost Considerations in Penile Implant Surgery from a Global Perspective

**DOI:** 10.1007/s11934-026-01324-5

**Published:** 2026-02-19

**Authors:** Kristian Black, Leila Momtazi-Mar, Hannah Glick, Scott D. Lundy, Raevti Bole, Petar Bajic

**Affiliations:** 1 Glickman Urological Institute, Cleveland Clinic, 9500 Euclid Ave, Cleveland, OH 44195-0001 USA; 2 Cleveland Clinic Lerner College of Medicine at Case Western Reserve University, Cleveland, OH USA; 3 Department of Urology, Michigan Medicine, Ann Arbor, MI USA

**Keywords:** Penile prosthesis, Erectile dysfunction, Cost-effectiveness, Insurance coverage, Ambulatory surgery, Global health disparities

## Abstract

**Purpose of Review:**

This review evaluates global trends, economic considerations, and recent innovations in penile prosthesis surgery for erectile dysfunction (ED). It aims to examine rising procedural costs, disparities in accessibility, and strategies to enhance affordability particularly in low- and middle-income countries (LMICs).

**Recent Findings:**

Despite its cost-effectiveness and high patient satisfaction, penile prosthesis surgery remains underutilized due to increasing costs and inconsistent insurance coverage. In the U.S., a shift toward ambulatory surgical centers may help reduce procedural costs. Low-cost devices like the Shah Malleable Prosthesis have improved accessibility in LMICs but face limited global adoption due to regulatory and evidence gaps. Broader health system reforms and novel tools such as predictive digital models may further mitigate economic barriers.

**Summary:**

Penile prosthesis implantation is a durable and effective treatment for ED but remains financially inaccessible to many patients worldwide. Reducing costs through outpatient surgery, insurance expansion, and validated low-cost devices can improve global access. Future progress requires coordinated policy reform and investment in scalable, equitable care pathways.

## Introduction

Erectile dysfunction (ED) is the persistent inability to achieve and/or maintain an erection sufficient for satisfactory sexual performance [1]. The global prevalence of ED ranges widely, from 13% to 71%, but it is estimated to affect at least 1 in 8 men and potentially as many as 3 in 4 men.

Penile prosthesis implantation is the gold standard treatment for erectile dysfunction refractory to more conservative therapies. Despite increasing utilization, limited data suggest that the overall costs associated with penile prosthesis surgery have substantially increased over time, potentially creating barriers to access [2]. In many countries, penile prostheses are not covered by national health systems, making these rising costs especially burdensome. As a result, many ED treatments—including penile prostheses—remain financially inaccessible to a large proportion of the global population [3]. This review evaluates recent trends, economic analyses, and emerging innovations in penile prosthesis surgery from an international perspective.

## Global Trends in Penile Prosthesis Surgery

Measured by preserved quality of life years, penile prosthesis implantation is the most cost-effective treatment for ED following failed PDE5i therapy [4]. In fact, Shauly et al. found that the cost of lifetime medical therapy with PDE5i was over $15,000 more than surgery, even when adjusted for inflation.

In the United States, most implants performed are three-piece inflatable penile prostheses (IPPs). The ratio of IPP to malleable penile prosthesis (MPP) placement increased from 2.3:1 in 2003 to 25:1 by 2012 and has remained stable since [2, 5]. Similarly, IPPs are more commonly used than MPPs in many European countries. However, international trends vary, with MPPs preferred in countries such as Saudi Arabia, India, and Brazil—likely due to cost and lack of national health plan coverage [2].

Currently, the most widely used inflatable penile prostheses (IPPs) are produced by two manufacturers: Boston Scientific, which offers the three-piece AMS 700™ and Coloplast, which produces the three-piece Titan^®^. Additional inflatable devices are available from other manufacturers, such as the Rigicon Infla10^®^ and Zephyr’s three-piece ZSI 475 and its gender-affirming variant, the ZSI 475 FTM. For malleable penile prostheses (MPPs), the Boston Scientific Tactra^®^ and Coloplast Genesis^®^ are most commonly implanted in the United States. However, several other malleable devices—including the Rigicon Rigi10^®^, Zephyr ZSI 100^®^, Promedon TUBE^®^, and the low-cost Shah Malleable—are available in select domestic and international markets.

While both MPPs and IPPs have evolved to improve reliability and reduce infection risk, their associated costs have also risen. Device prices have increased proportionally with inflation, but inclusive surgical costs have outpaced inflation by nearly a factor of four, according to a 2022 systematic review. These findings are limited by the predominance of data from the United States and Europe and the absence of studies published after 2014 [2].

In many low- and middle-income countries (LMICs), the cost of ED therapies is disproportionately high relative to income, placing these treatments beyond the reach of much of the global population outside of insurance coverage. Shah et al. compared the per capita nominal gross domestic product (GDP) versus the cost of various ED treatments in USD. In India, the nominal GDP per capita in 2019 was $2,104, while the cost of MPP was $1,600 versus $10,000 for an IPP. Similarly, in Egypt, the nominal GDP per capita in 2019 was $3,020, while the cost of MPP was $850 and IPP was $5,700. For context, in 2019 the US per capita GDP was approximately $65,000 [3]. These stark disparities illustrate how the high cost of ED treatments relative to income creates substantial barriers to access in resource-limited settings.

### Insurance Coverage and Accessibility

In the US, most commercial insurance companies (Blue Cross Blue Shield, Aetna, Anthem, Cigna, HCSC) and government-funded health programs (Medicare, Medicare Advantage, Tricare, and Veterans Affairs) have coverage policies for treatment of ED, deeming it medically necessary [1, 6, 7]. However, coverage for ED therapies varies widely by treatment type, state, and insurance policy.

A 2021 state-based Medicaid analysis found coverage for penile prosthesis placement existed in 28 states, but that there was a considerable lack of transparency on who could qualify for coverage or prior authorization criteria [8]. When assessing insurance coverage for IPP placement in the United States, Khera et al. found that roughly 80% of their cohort had coverage for IPP implantation. A recent Medicare cost-modeling analysis found that ED therapies—including IPP—recommended more strongly by AUA guidelines were associated with lower out-of-pocket costs for patients [9].

In the US, much of the variation in coverage for management of ED and IPP implantation comes with employer-sponsored health insurance plans [10, 11]. The most common reason for lack of coverage for IPP placement in the US is employer exclusion, which has increased by nearly 30% between 2013 and 2021 despite rising ED prevalence during the same period [6, 12–14].

Failure to cover ED treatments results in significant indirect costs. In the U.S., untreated ED among working-age men with employer-sponsored insurance accounts for an estimated $9.3 billion in lost productivity annually [15]. In this study, men with ED had an additional 280 h/year of work impairment as opposed to men without ED – similar to workdays lost to diseases such as diabetes, cardiovascular disease, and stroke. Similarly, a global study by Goldstein et al. evaluating productivity and absenteeism across eight countries (Brazil, China, France, Germany, Italy, Spain, the UK and the US) showed that men with ED had significantly higher rates of absenteeism and work productivity impairment [16].

These indirect losses may be even more severe in LMICs, where labor tends to be more physically demanding. Expanding insurance coverage to include ED treatments—particularly penile prosthetics—can yield not only individual benefits but also broader economic returns through restored productivity and improved psychosocial well-being as ED is closely associated with mental health conditions such as depression, anxiety, and diminished quality of life [11, 15–19].

Globally, accessibility to ED treatment or penile prosthesis implantation varies at least in part due to the different health coverage systems which exist. As mentioned above, the mixed public and private system in the US creates barriers for patients to achieve coverage, especially with employer-sponsored plans that can exclude certain treatment modalities, such as IPP. Similar to the US, coverage for IPP placement for ED globally may depend on what is deemed medically necessary. A survey of Canadian urologists showed the variable nature of coverage across the country [20]. Continued measures to decrease the cost of prosthesis implantation (via lower cost devices, or strategies such as outpatient surgery) along with advocacy and policy change to increase coverage for ED treatments are crucial to decrease barriers and increase access to care for patients globally [2, 18, 21, 22].

## The Role of Ambulatory Surgery in Cost Curtailment

In the US, there has been a dramatic shift towards penile prothesis implantation in outpatient settings [2]. Based on Healthcare Cost and Utilization Project data from 2016 to 2019, a total of 67,722 penile prostheses were implanted in the United States, with 93.1% performed in ambulatory surgery centers (ASCs). The average estimated facility charge for ASC procedures was approximately half that of inpatient placements ($25,935 vs. $51,594; *p* < 0.01) [23]. While this observed difference in facility charges suggests that ASC utilization may represent a promising avenue for reducing expenditures associated with surgical management of ED, these findings are based on data that have not yet undergone peer-reviewed validation and should therefore be interpreted with appropriate caution.

Similarly, a 2014 cohort study from two U.S. states showed lower procedural and 30-day acute care costs for surgeries performed in ambulatory versus inpatient settings, with no difference in 30-day revisit rates [24]. These findings support the safety and feasibility of IPP placement in ambulatory settings. To optimize the use of ambulatory surgical centers for penile prosthetics, specific workflows should be developed to determine patient eligibility to maximize safety [25]. For example, if the underinsured are less likely to receive care in an ambulatory setting due to comorbidities, workflows to mitigate risk for various comorbid diseases might be employed to support transitioning to ambulatory care for these higher risk patients.

Another study in the United States modeled the economic impact of reduced postoperative visits for patients who received IPP. The authors concluded that fewer post-operative visits could increase appointment availability for other patients, resulting in additional insurance reimbursements by recapturing clinician time that can be reallocated to other care (e.g., vasectomy, new ED evaluations). For example, if a surgeon performs 100 implants yearly, the recaptured time in their clinical schedule could enable 37 additional in-office vasectomies, translating to $12,234 in reimbursement [26]. These findings mirror current trends of surgeons increasingly using nurses and advanced practice providers (APPs) to manage routine post-operative visits, though the safety of significantly reducing surgeon-led follow-up requires further study.

Outside of the United States, a study conducted in Spain using a cost-consequence model estimated that outpatient penile prosthetic implantation resulted in savings of €962 per patient plus one hospital bed day per procedure. At the time of the study, only 8.3% of implants were implanted in the outpatient setting, and the authors estimated that if the percentage increased to 50% or 70%, this would result in annual savings of €279,657 and €413,785, respectively [21]. More studies, particularly outside of the United States, are needed to confirm the safety and cost savings of ambulatory surgery internationally to improve confidence of healthcare providers to adopt these practices globally.

## Innovations and Low-Cost Options

While MPPs are generally more affordable than IPPs, the Shah Malleable Prosthesis (Surgiwear, Uttar Pradesh, India) stands out as an ultra-low-cost model that has largely dominated the Indian market since its introduction in 1996 [27]. The Shah malleable prosthesis is constructed entirely of silicone and incorporates segmental rigidity to provide both axial stiffness and a hinge for concealment. Length can be modified by trimming the proximal segment or adding rear tip extenders, while girth can be reduced by removing outer silicone sleeves, allowing intraoperative tailoring to a wide range of corporal dimensions. This high degree of adjustability, combined with single-material construction and the absence of complex interchangeable components, reduces manufacturing costs and simplifies distribution, making the device particularly well suited for use in low- and middle-income countries where streamlined production and supply-chain efficiency are critical [28].

In India the Shah prothesis retails for approximately $300 USD, making it substantially more affordable than Western alternatives such as the Tactra prosthesis (Boston Scientific, Marlborough, MA, USA), which is priced around $1,800 USD [22]. In addition to these interbrand differences, substantial intrabrand international cost variability also exists [3]. For example, the Tactra prosthesis is priced at $850 USD in Egypt—28% of the country’s GDP per capita—yet rises to $4,300 USD in Indonesia, equivalent to 104% of GDP per capita. These discrepancies likely reflect variation in value-added taxes (VAT), procurement structures, and distribution systems rather than intrinsic device costs. Because device-level invoices are not publicly available, country-level pricing differences are best interpreted in the context of these broader macroeconomic factors. Nevertheless, such disparities constitute major barriers to access, particularly in LMICs, where device prices far exceed average incomes and render IPPs inaccessible to most patients. Designing simpler implants that could be produced locally in markets such as Indonesia may help reduce these financial burdens.

Despite its significantly lower price point, the Shah MPP has demonstrated favorable outcomes in terms of both patient satisfaction and safety. Krishnappa et al. reported that 44 of 52 patients (85%) selected the highest possible response in the “satisfaction” domain of the validated Erectile Dysfunction Inventory of Treatment Satisfaction (EDITS), including both retrospective patients assessed 2–5 years after surgery (*n* = 26) and prospective patients assessed at 6 months (*n* = 26). Additionally, minor (21%) and major complications (11%) were comparable to those seen in larger studies of legacy MPP, including the Spectra and AMS 600/650 devices [22, 29, 30].

However, broader international adoption of low-cost alternatives such as the Shah MPP remains constrained by systemic barriers—chief among them being the lack of investment in high-quality clinical trials necessary to establish safety, efficacy, and long-term outcomes. Regulatory challenges, including the substantial costs of obtaining Food and Drug Administration (FDA) approval—estimated at nearly $570,000 in user and annual fees for Class III devices such as the Coloplast Titan or AMS 700, excluding clinical trial and development expenses—further hinder entry into the U.S. market [31].

To date, the study by Krishnappa et al. remains the only report presenting Shah-specific outcome data, underscoring the limited evidence base for this low-cost alternative [22]. Other publications—such as the semiurban Indian cohort by Patil et al. [32], the Turkish single-institution series by Bozkurt et al. [33], isolated case reports in reconstructive settings [34, 35], and narrative reviews of malleable devices [27, 28]—include the Shah prosthesis but do not stratify outcomes by device. In the absence of robust, device-specific evidence, low-cost prostheses like the Shah are rarely incorporated into clinical guidelines or highlighted in international forums, limiting their visibility and acceptance among prosthetic surgeons.

## Alternative Cost-Reducing Strategies

Screening for cardiovascular disease (CVD) and metabolic syndrome (MetS), along with lifestyle changes to mitigate those conditions, represents a cost-efficient and preventative approach to preventing ED—now widely recognized as an early clinical marker of systemic vascular dysfunction. Identifying and managing risk factors such as hypertension, dyslipidemia, diabetes, and obesity, along with smoking cessation, not only prevents or delays ED but also reduces its global economic burden [36, 37].

Emerging tools such as large language models (LLMs) offer low-cost, scalable solutions for ED risk stratification using minimal clinical data and patient-reported outcomes [38]. However, the utility of LLMs in LMICs is currently limited by the lack of digital health infrastructure, centralized health registries, and data interoperability. While such predictive models show promise, investment in digital health systems is necessary to realize their full potential in under-resourced settings.

## Conclusion

Penile prosthesis surgery remains a highly effective, durable, and patient-satisfying treatment for erectile dysfunction, yet access to this intervention is hindered globally by rising procedural costs, limited insurance coverage, and health system disparities. The increasing shift toward outpatient surgical settings and the development of low-cost prosthesis models—such as the Shah implant—demonstrate promising strategies for cost containment. Likewise, preventative health measures, digital tools for risk prediction, and broader insurance inclusion have the potential to mitigate both the direct and indirect costs of ED. As the global burden of erectile dysfunction grows, so too must efforts to implement equitable, economically sustainable pathways to care, particularly in LMICs. Multinational collaboration, policy reform, and targeted investment will be essential to ensure that penile prosthesis surgery becomes an accessible and affordable option for all patients in need.

## Key References

- Gill B, Huang J, Wilson C, et al. Economic impact of reduced postoperative visits after inflatable penile prosthesis implantation. J Comp Eff Res. 2025;14:e240204.
  - ○ Quantifies the downstream financial benefits of reduced follow-up burden after IPP, supporting efficiency in care delivery.

- Allen MS, Wood AM, Sheffield D. The Psychology of Erectile Dysfunction. Curr Dir Psychol Sci. 2023;32(6):487–493.
  - ○ Emphasizes the psychosocial burden of ED and its systemic consequences, framing the urgency for broader treatment access.

- Krishnappa P, Tripathi A, Shah R. Surgical Outcomes and Patient Satisfaction With the Low-Cost, Semi-Rigid Shah Penile Prosthesis: A Boon to the Developing Countries. Sex Med. 2021;9(6):100399.
  - ○Only published study reporting Shah-specific outcome data; demonstrates strong patient satisfaction and favorable complication profile at a low cost.

- Khera M, Le B, DaJusta D, et al. Implantable Penile Prosthesis for Erectile Dysfunction: Insurance Coverage in the United States. Urol Pract. 2023;10(6):501–510.
  - ○ Provides a contemporary look at insurance coverage trends for IPPs in the U.S., highlighting the role of policy in access to care.

- Brown W, Gill B, Gorbatiy V, et al. Trends and Costs of Ambulatory vs Inpatient Penile Prosthesis Placement. J Sex Med. 2023;20(supplement_4):qdad060.446.
  - ○Examines the dramatic cost savings associated with outpatient IPP placement, a key economic strategy discussed in the review.

- Torremadé J, Moncada I, Heras M, et al. Cost-consequence analysis of penile prosthesis implantation in outpatient setting in Spain. Int J Healthc Manag. 2024;17(2):160–167.
  - ○ Provides rare non-U.S. data on cost-effectiveness of outpatient IPP, reinforcing the global applicability of this strategy.

## Data Availability

No datasets were generated or analysed during the current study.
